# ^177^Lu-OPS201 targeting somatostatin receptors: in vivo biodistribution and dosimetry in a pig model

**DOI:** 10.1186/s13550-016-0204-9

**Published:** 2016-06-13

**Authors:** Seval Beykan, Jan S. Dam, Uta Eberlein, Jens Kaufmann, Benedict Kjærgaard, Lars Jødal, Hakim Bouterfa, Romain Bejot, Michael Lassmann, Svend Borup Jensen

**Affiliations:** Department of Nuclear Medicine, University of Würzburg, Oberdürrbacher Str. 6, 97080 Würzburg, Germany; Department of Nuclear Medicine, Aalborg University Hospital, Aalborg, Denmark; Octreopharm Science GmbH, Ipsen Group, Berlin, Germany; Biomedical Research Laboratory, Department of Clinical Medicine, Aalborg University Hospital, Aalborg, Denmark; Department of Chemistry and Biochemistry, Aalborg University, Aalborg, Denmark

**Keywords:** Neuroendocrine tumor (NET), Dosimetry, Antagonist, JR11, OPS201, Pig model, Lutetium-177, PRRT

## Abstract

**Background:**

^177^Lu is used in peptide receptor radionuclide therapies for the treatment of neuroendocrine tumors. Based on the recent literature, SST2 antagonists are superior to agonists in tumor uptake. The compound OPS201 is the novel somatostatin antagonist showing the highest SST2 affinity. The aim of this study was to measure the in vivo biodistribution and dosimetry of ^177^Lu-OPS201 in five anesthetized Danish Landrace pigs as an appropriate substitute for humans to quantitatively assess the absorbed doses for future clinical applications.

**Results:**

^177^Lu-OPS201 was obtained with a specific activity ranging from 10 to 17 MBq/μg. Prior to administration, the radiochemical purity was measured as *s* > 99.7 % in all cases. After injection, fast clearance of the compound from the blood stream was observed. Less than 5 % of the injected activity was presented in blood 10 min after injection. A series of SPECT/CT and whole-body scans conducted until 10 days after intravenous injection showed uptake mostly in the liver, spine, and kidneys. There was no visible uptake in the spleen. Blood samples were taken to determine the time-activity curve in the blood. Time-activity curves and time-integrated activity coefficients were calculated for the organs showing visible uptake. Based on these data, the absorbed organ dose coefficients for a 70-kg patient were calculated with OLINDA/EXM. For humans after an injection of 5 GBq ^177^Lu-OPS201, the highest predicted absorbed doses are obtained for the kidneys (13.7 Gy), the osteogenic cells (3.9 Gy), the urinary bladder wall (1.8 Gy), and the liver (1.0 Gy). No metabolites of ^177^Lu-OPS201 were found by radio HPLC analysis. None of the absorbed doses calculated will exceed organ toxicity levels.

**Conclusions:**

The ^177^Lu-OPS201 was well tolerated and caused no abnormal physiological or behavioral signs. In vivo distributions and absorbed doses of pigs are comparable to those observed in other publications. According to the biodistribution data in pigs, presented in this work, the expected radiation exposure in humans will be within the acceptable range.

**Electronic supplementary material:**

The online version of this article (doi:10.1186/s13550-016-0204-9) contains supplementary material, which is available to authorized users.

## Background

The radionuclide ^177^Lu (Lutetium-177) is being increasingly used in targeted radionuclide therapy (TRT) because of its favorable decay characteristics and the possibility of reliable labeling of biomolecules used for tumor targeting. Initially, ^177^Lu was used in a colloidal form for interstitial injections for intraperitoneal radioimmunotherapy of ovarian cancer [1]. Furthermore, ^177^Lu is increasingly used in radioimmunotherapy (RIT) clinical trials to label different kinds of monoclonal antibodies [2]. Presently, the most frequent clinical use of ^177^Lu is in peptide receptor radionuclide therapy (PRRT) for treatment of neuroendocrine tumors (NETs), in some cases parallel to or in conjunction with ^90^Y [3–7].

The radiolabeled somatostatin receptor (SST2) agonists DOTA-[Tyr^3^]octreotate (DOTATATE), DOTA-[Tyr^3^]octreotide (DOTATOC), and DOTA-[NaI3]octreotide (DOTANOC), as well as the antagonists OPS201^1^ and OPS202^2^ are successfully implemented in the clinical routine for imaging and treatment of NETs which are overexpressing the somatostatin receptor SST2. The peptidic vector connects directly to the relevant receptors, and consequently, ^111^In-radiolabeled octreotide was used firstly for diagnostic purposes [8] whereas the combination with ^177^Lu or ^90^Y is used for PRRT [5, 9]. Currently, for the diagnosis and staging of NETs, ^68^Ga-radiolabeled DOTA-somatostatin analogues combined with PET/computed tomography (CT) is considered as state of the art. However, due to the short half-life of ^68^Ga (1.13 h), a pretherapeutic dose assessment is challenging. In addition, the use of different radiopharmaceuticals in therapy and diagnostics may cause misestimating. Therefore, further correlation studies are necessary.

The application of SST2 agonists for TRT involves the systemic administration of a radiolabeled peptide designed to target overexpressed receptors on tumor cells with high affinity and specificity [5, 7, 10, 11]. Contrary to the paradigm that internalization and the resulting accumulation of radiotracers in cells is necessary for efficient tumor targeting, recent preclinical and clinical studies have indicated that antagonists are superior to the agonist especially for tumor targeting despite little to no internalization in cells [7, 10, 12]. OPS201 is a novel SST2 antagonist which, to the best of our knowledge, has the highest tumor to organ dose ratios and tumor detection comparied to other SST2 antagonists [13]. Radiolabeled SST2 antagonists are not yet well established for tumor targeting mainly because they do not internalize into tumor cells [10].

However, based on the recent in vitro preclinical and human studies, it was shown that the uptake of SST2 antagonists is higher compared to SST2 agonists [10, 11, 13, 14], although the absorbed dose to the kidneys, the main organ at risk after treatment of NETs with DOTA-labeled compounds, was around 50 % higher for the antagonist as compared to the agonist ^177^Lu-octreotate [2].

For assessing the absorbed doses in humans in potential clinical applications, guidelines require the investigation of the biodistribution of ^177^Lu-OPS201 in an animal model. Since pigs show similar biodistribution and physical properties to humans and because their sizes fit a human SPECT/CT scanner, they were chosen in this study to determine the effect of the radiotracer on the kidneys and other organs at risk. In addition, pig studies have the advantage of long follow-up times similar to human studies.

Up to now, one clinical human study and one preclinical mouse model study have been conducted with ^177^Lu-DOTA-JR11 (OPS201) [10, 12]. In the first human pilot study with ^177^Lu-DOTA-JR11 (OPS201) by Wild et al. [10], the efficacy of ^177^Lu-OPS201 was demonstrated by therapy response. The patients in this study already had grade 2 or 3 chronic renal failure. A sixfold higher tumor-to-kidney ratio was observed for ^177^Lu-DOTA-JR11 (OPS201) compared to ^177^Lu-DOTA-octreotate in spite of 1.5 times higher kidney doses for ^177^Lu-DOTA-JR11 (OPS201). The significant uptake of ^177^Lu-DOTA-octreotate in the kidneys requires supplemental investigation. In addition, comparable results to the pilot human study were reported in the mouse model study by Dalm et al. [12]. In this study, mice received one therapeutic injection of ^177^Lu-DOTA-JR11 (OPS201) and experiments were done in a rather short follow-up time especially for quantification of the biodistribution and dosimetry [2].

Therefore, the aim of the present study was to measure the biodistribution of ^177^Lu-OPS201 in pigs as a model to quantitatively assess the absorbed doses in humans in potential clinical applications. Pigs were chosen as they mimic humans’ physiology better than small animals such as mice or rats. In addition, pigs allow for multiple blood samples as well as dosimetry assessment during a long period.

## Methods

### Physical properties of ^177^Lu

The radionuclide ^177^Lu has a 6.647-day half-life and disintegrates by beta-emission (probability, 100 %) to the ground state and to the three excited levels of ^177^Hf. The maximum beta energy is 498 keV. There are two gamma emissions that have 208.3 keV photon energies with a probability of 10.38 % per disintegration and 112.9 keV (6.2 %). ^177^Lu-labeled compounds show many advantages for dosimetry assessments due to attractive physical properties which comprise low abundance of photons for sufficient post-therapy imaging, a clearly separated gamma peak at 208.3 keV, and a low range of beta particles. Due to its low photon abundance of emission probability, even with a 7 GBq amount of activity, it allows the post-therapeutic imaging with avoiding camera dead-time related effects which is only possible with high activities [2].

### Labeling of ^177^Lu-OPS201

^177^Lu-OPS201 was produced on an automated cassette-based synthesis system (E&Z Modular-Lab PharmTracer). The OPS201 precursor (24 nmol, Octreopharm Sciences GmbH) was reacted with a non-carrier added (n.c.a.) ^177^LuCl_3_ (Isotope Technologies Garching GmbH (ITG)) in a sodium acetate/ascorbate buffer (pH 4.5) at 80 °C for 20 min, subsequently purified by C18 SPE, was reformulated in an ethanol-saline solution and sterile filtered. The radiolabeled product was stored overnight at 4 °C. The ready-to-inject formulation was allowed to equilibrate to room temperature for a minimum of 1 h before administration. Quality control of the radiolabeled test substance was performed after the synthesis and immediately before administration to confirm its stability.

### Animal study

For analyzing the biodistribution and internal dosimetry of ^177^Lu-labeled peptides in preclinical and clinical studies, the mean activity 105 MBq (97–113 MBq) ^177^Lu-OPS201 were injected to five anesthetized Danish Landrace pigs (three females and two males, age 3 months, weight 25–32 kg). All animals originated from a specific-pathogen-free farm, where all animals were regularly tested for infectious agents (bacteria and virus).

The animals underwent up to 7 days of acclimatization prior to study start. The body weights of the animals were recorded. All animals were confirmed to be in good health.

Anesthesia was induced with Zoletil 50 Vet mixture (a mixture of two dissociative anesthetics (ketamine 6.25 mg/mL and tiletamine 6.25 mg/mL), a benzodiazepine (zolazepam 6.25 mg/mL), a synthetic opioid (butorphanol 1.25 mg/mL), and xylazine (6.5 mg/mL), an alpha 2 adrenergic agonist, containing sedative, hypnotic, analgesic, and muscle-relaxing properties). Anesthesia was maintained with continuous intravenous infusions of midazolam and fentanyl based on the clinical demand.

After anesthesia, a cuffed Charriere 6.5 endotracheal tube was inserted (Potex, Smiths Medical, Watford, UK) and connected to an Oxylog® 2000 ventilator (Dräger Medical GmbH, Lübeck, Germany). Normoventilation was obtained using a volume controlled ventilation mode with tidal volumes of 6–8 mL/kg, respiratory frequency of 12–14/min, and positive end expiratory pressure (PEEP) of 5 cm H_2_O. The settings were adjusted to the level of end-tidal CO_2_. The exact levels of pH, CO_2_, and potassium were tested in arterial blood samples (ABL 800, Radiometer, Copenhagen, Denmark). The inspired oxygen fraction was 60 %. The animals were protected with blankets against hypothermia.

A 7-French central venous catheter was inserted into a jugular vein for medications and fluid. A 5-French catheter was inserted in the femoral artery for blood pressure monitoring and arterial blood sampling. Continuous blood pressure and ECG were monitored using Datex-Ohmeda S/5 (GE Healthcare, Broendby, Denmark). A bladder catheter was inserted for continuous urinary drainage except in pig 4. All pigs, except pig 4, received an intravenous amino acid infusion (28.5 g/L L-arginine·HCl, 29.3 g/L L-lysine·HCl, 2.7 g/L NaCl) for approximately 4 h, starting approximately 1 h prior to the intravenous injection of ^177^Lu-OPS201. Since the catheter could not be inserted for pig 4 and any urinary drainage during the 4 h amino acid infusion combining with ^177^Lu-OPS201 would directly cause ^177^Lu related radiation contamination of the scanner, no amino acid was administered to pig 4. Although in normal clinical case all patients will receive an amino acid infusion, we assessed to investigate the impact of amino acid infusion on the absorbed doses. On the following days 2, 4, and 6, no arterial line was inserted. Blood samples were taken from the ear.

### Blood and urine sampling and processing

After administration, 12 arterial blood samples were taken at nominal time points of 0.5, 1, 2, 5, 10, 20, 30, 50, 75, 100, 200, and 300 min to determine the time-activity curve of the blood. In addition, three venous blood samples were drawn at 2, 4, 6, and 10 or 12 days after injection. The blood samples at 30, 50, 100, 200, and 300 min and 2, 4, 6, and 10 or 12 days were used for metabolite analysis. From the whole blood samples, 1 mL of the whole blood was transferred into a counting tube, and the rest was centrifuged to obtain plasma. From the plasma, 1 mL was transferred into a counting tube. Both sets of tubes (whole blood and plasma) were analyzed in a gamma counter normalized to measure ^177^Lu-OPS201. All samples were measured, and the activity was decay-corrected to the time of sampling. Plasma samples (1 mL) were denatured with acetonitrile (1 mL) and centrifuged, and the supernatant (0.5 mL) was diluted with water (1 mL) for HPLC (high-performance liquid chromatography) analysis. A reference sample in the form of non-radioactive OPS201 was eventually added, and 1 mL of the (spiked) solution was transferred into the HPLC loop for analysis.

Urine was collected for up to 4 h post-injection of ^177^Lu-OPS201, using a urine catheter. The weight of the collected urine was measured, and three 1 mL aliquots of the urine sample were analyzed in a gamma counter (PerkinElmer, 2480 Wizard). Urine samples (1 mL) were analyzed directly by HPLC.

### Imaging and reconstruction

SPECT/CT data and whole-body (WB) planar images were acquired on a SIEMENS Symbia T16 (SIEMENS AG). On day 0, three WB and SPECT/CT scans were performed in the first 3 h. Further scans were done at 2, 4, 6, and 12 days (pigs 1 and 2) and at 2, 4, and 10 days (pigs 3–5) after administration of approximately 105 MBq ^177^Lu-OPS201. The acquisition duration was 50 min for all nuclear medicine scans: 10 min for whole-body and 40 min whole-body SPECT (2 bed positions of 20 min each). In addition, a 5 min CT was performed for attenuation correction.

The physiological parameters of the animals were monitored and, if necessary, adjusted during the scans. For reconstruction, CT-based attenuation correction and triple-energy-window based scatter corrections were applied. The images were reconstructed with the FLASH-3D iterative reconstruction algorithm with six iterations and six subsets. The resulting images were smoothed with a 6 mm Gauss filter.

### Dosimetry analysis

#### Quantification of activity in VOIs and integration of the time-activity curves

**Fig. 1:**
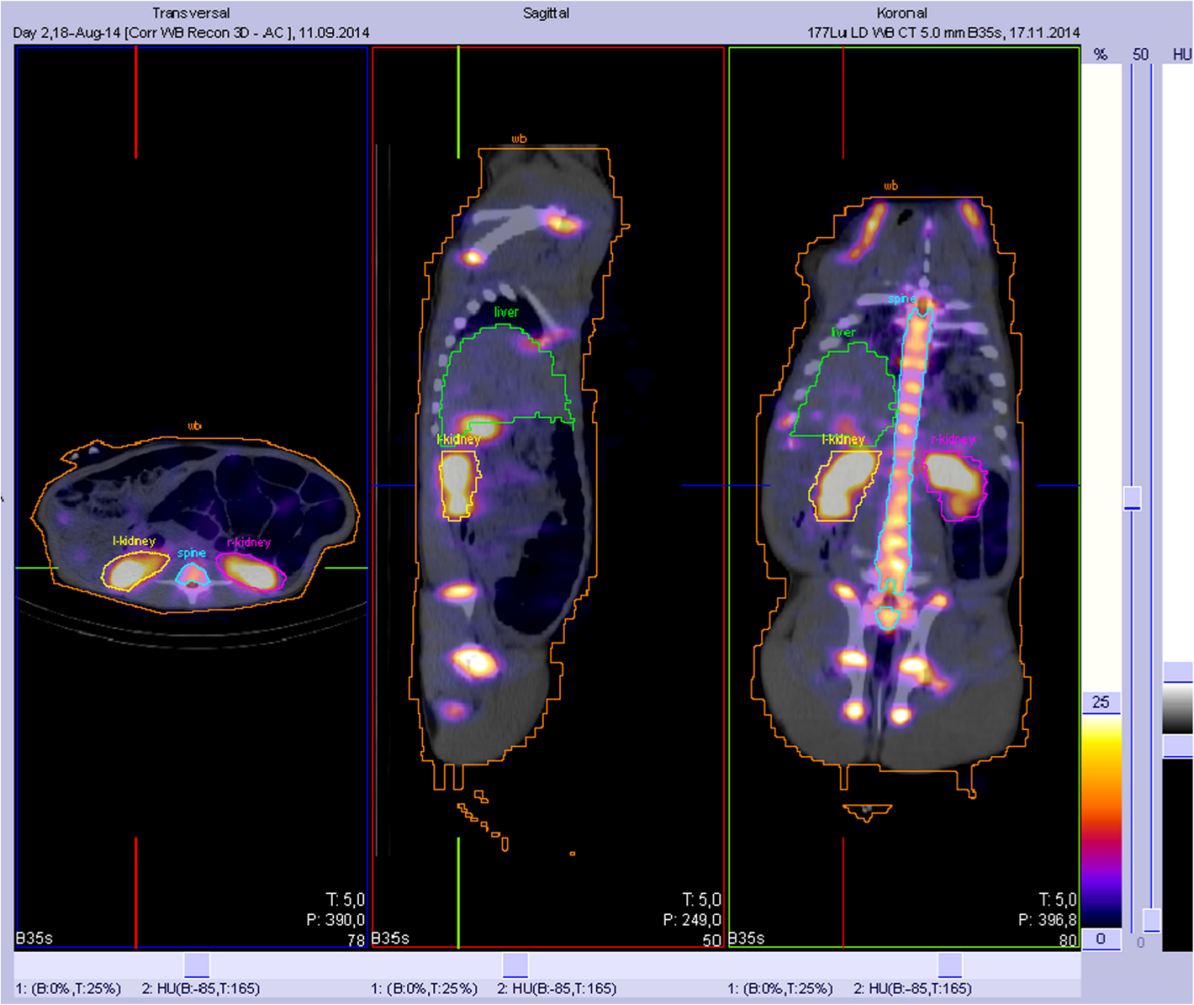
Three-view display of the VOI analysis for the selected organs (pig 2, day 2)

To quantify the amount of activity, the average percentage values corresponding to the injected radioactivity (%A) per organ as a function of time were calculated for the heart, liver, right kidney, left kidney, bladder, spine, and WB for each pig via a manual volume of interest (VOI) analysis (see Fig. 1). All VOIs were drawn based on the CT scan. In order to avoid spill-out effects, CT-based organ VOIs were enlarged as matching 2 voxel plus their actual CT-based volumes. The whole-body uptake of the first scan was set to 100 %. The time-activity curves (TACs) for the blood were analyzed separately from the collected samples, and the blood time-activity curves (decay-corrected to the time of injection) were fitted using Graph Pad Prism (6.07). Blood-based dosimetry as a surrogate for the bone marrow was performed according to the Shen method [15].

For internal dosimetry, the organ-specific time-integrated activity coefficients (TIACs) were calculated by integration of the respective TACs for each organ and blood using the software solution NUKFIT [16], choosing the optimal fit functions as proposed by the code. The TIAC is estimated by analytically integrating the fitted functions. Its standard error is determined assuming Gaussian error propagation. For this investigation, a systematic error in activity quantification of 10 % was assumed.

The TIACs of the urinary bladder contents were integrated by using a trapezoidal integration method and assuming physical decay after the last data point. The TIAC of bone was calculated based on the approach that 19 % of the total skeleton mass is considered as the mass of the spine [10]. No inter-species scaling was performed while calculating the TIACs and TACs.

By using the values of the TIACs for a selected group of organs, the absorbed dose coefficients for a male patient were calculated by using OLINDA/EXM [17].

## Results

### Labeling of ^177^Lu-OPS201

^177^Lu-OPS201 was obtained in three batches with a specific activity ranging from 10 to 17 MBq/μg. The radiochemical purity was determined by reversed phase HPLC at the end of synthesis (EOS) and just prior to administration to be always >99.7 %. The values of the blood and plasma activity based on the serial blood samples collected for up to 12 days post-dosing, expressed in Bq/mL (decay-corrected to the time of sampling) as well as in %IA/mL (percentage injected activity per volume of blood, decay-corrected to the time of injection) is provided in Additional file 1: Table S1. The results of denatured plasma samples analyzed by HPLC for the detection of radioactive metabolites are also shown in Additional file 1: Table S2). No metabolites could be detected. During the 4 hours of arginine infusion plasma, potassium rose in all pigs from normal values (3.9–4.1 mmol/L up to 7.0 mmol/L); however, unexpectedly, it also rose to 6.6 mmol/L in pig 4 that did not receive arginine for renal protection. On the next day, all pigs had normal values of potassium. Hypothetically, the possible explanation associated with the rise in plasma potassium could be either impaired Na/K pump function or a partial blockage of oxygen to the cells resulting in a depression of the Na/K pump and the feeling of oxygen-lack triggered hyperventilation. In other words, the rise in plasma potassium level may be highly related to stress.

### Biodistribution and dosimetric calculations

**Fig. 2:**
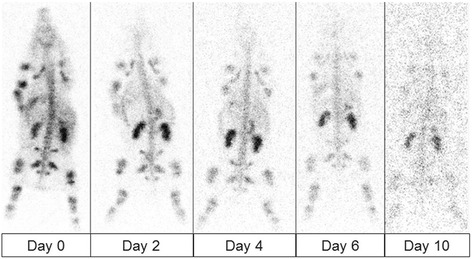
The posterior whole-body scans of pig 1 at the subsequent days after injection of ^177^Lu-OPS201. Scan duration of WB planar images was 10 min. The image quality of WB planar scans with ^177^Lu-OPS201 was high; kidney, spine, and bone uptake were easily distinguished in all days of WB planar scan

**Fig. 3:**
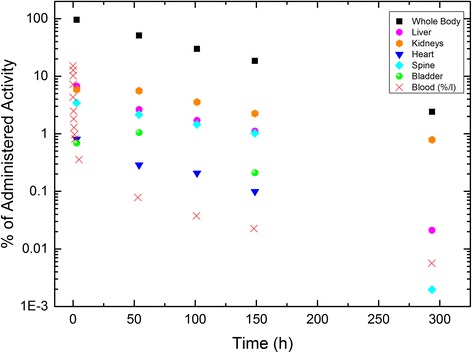
Time-activity curves of the selected organs in pig 1 after an injection of 107 MBq ^177^Lu-OPS201. The uptake in the blood is given in %/L. At day 0, three WB and SPECT\CT scans were performed in the first 3 h. For better visualization and since the percentage activity values of three scans in day 0 were quite similar for the selected organs, we show only the data of scan 3 for day 0. Since the percentage administered activity value of the heart and bladder at days 3 and 12 is less than 1 × 10^−3^, they are not shown in the graph

The image quality of the WB planar scans of all pigs was sufficient for a dosimetric analysis (for pig 1 displayed in Fig. 2). Even after 10 days, there was still a detectable kidney uptake. Figure 3 shows the resulting TACs for the same animal (pig 1). The measured organ uptake values were shown in Additional file 1: Table S3. The organs with the highest uptake were the bone and kidneys, followed by the liver. The resulting TIACs are summarized in Table 1. The highest TIACs were observed for the bone, kidneys, and liver (mean TIAC_bone_, 11 ± 9.9 h; mean TIAC_kidney_, 9.4 ± 3.6 h; mean TIAC_liver_, 4.2 ± 1.2 h). There was no visible uptake in the spleen; however, the uptake in the kidneys, spine, and bone were easily distinguished in the WB planar scan on all the scan days (see Fig. 2). A fast clearance of the compound from the blood was observed in the first minutes after administration resulting in less than 5 % of the injected activity per liter of blood circulating 10 min after the injection. ^177^Lu-OPS201’s blood clearance followed a biexponential pattern with an initial rapid decrease (T_½_*α* = 2.0 ± 0.9 min; 80.1 ± 3.5 % clearance in the α phase), followed by an excretion-related blood clearance (T_½_*β* = 36 ± 13 min). In pigs infused with an amino acid solution (*n* = 4), 23.4 ± 3.5 % of the decay-corrected activity was excreted in the urine during 4 h post-injection. No metabolites of ^177^Lu-OPS201 were detected by radio HPLC analysis of the plasma and urine samples. The complete list of the corresponding mean absorbed doses is provided in Table 2. The highest absorbed dose coefficients were observed in the kidneys (2.73 mGy/MBq), the osteogenic cells (0.79 mGy/MBq), the urinary bladder wall (0.35 mGy/MBq), and the liver (0.20 mGy/MBq). The range of kidney absorbed doses for pigs 1–3 and 5 was 1.8–2.7 Gy/GBq, and the kidney dose value of pig 4 was 4.5 Gy/GBq most likely due to lack of amino acid infusion. The kidney absorbed dose value of pig 4 was not eliminated from the mean absorbed dose calculation in order to provide a conservative assessment of the absorbed dose to the kidneys. The separated absorbed doses for each pig related to the selected organs are shown in Table 2. None of the absorbed doses exceeded organ toxicity levels (23 Gy critical organ dose for kidneys [18]).

**Table 1:** Time-integrated activity coefficients (TIACs—unit, h) values for the selected organs of each pigs and respective standard deviations (SD)

Organs	Pig 1	Pig 2	Pig 3	Pig 4	Pig 5	Mean ± SD (*n* = 5)
Whole body	81.73	72.23	60.95	75.09	80.08	74.02 ± 8.24
Remainder	44.63	53.95	41.46	47.32	45.17	46.51 ± 4.66
Liver	4.98	3.48	2.66	4.51	5.57	4.24 ± 1.17
R. kidney	4.76	3.29	4.69	7.82	2.94	4.70 ± 1.93
L. kidney	4.57	4.18	4.11	7.59	3.14	4.72 ± 1.69
Kidneys^a^	9.34	7.47	8.80	15.40	6.07	9.42 ± 3.58^c^
Heart	0.53	3.89	0.41	0.79	0.65	1.25 ± 1.48
Bladder contents	0.90	0.43	0.02	5.27	0.51	1.43 ± 2.17
Spine	4.03	0.57	1.42	0.29	4.16	2.10 ± 1.87
Bone^b^	21.22	3.00	7.47	1.55	21.91	11.03 ± 9.86
Red marrow	0.12	0.01	0.13	0.22	0.18	0.13 ± 0.08

^a^Sum of both kidneys

^b^Derived from spine values (19 % of the total skeleton mass)

^c^Mean ± SD values for the kidneys without pig 4 values are 7.92 ± 1.46 h

**Table 2:** Absorbed doses (mGy/MBq) obtained by using the TIACs of the considered organs and respective standard deviations (SD)

Target Organ	Pig 1	Pig 2	Pig 3	Pig 4	Pig 5	Mean ± SD (*n* = 5)
Adrenals	6.82E−02	7.69E−02	6.12E−02	7.39E−02	6.69E−02	6.94E−02 ± 0.01
Brain	5.80E−02	6.74E−02	5.24E−02	5.90E−02	5.88E−02	5.91E−02 ± 0.01
Breasts	5.59E−02	6.74E−02	5.14E−02	5.87E−02	5.66E−02	5.80E−02 ± 0.01
Gallbladder wall	6.61E−02	7.55E−02	5.91E−02	7.08E−02	6.61E−02	6.75E−02 ± 0.01
LLI wall	6.06E−02	7.10E−02	5.49E−02	6.55E−02	6.10E−02	6.26E−02 ± 0.01
Small intestine	6.20E−02	7.25E−02	5.65E−02	6.65E−02	6.21E−02	6.39E−02 ± 0.01
Stomach wall	6.13E−02	7.23E−02	5.61E−02	6.55E−02	6.13E−02	6.33E−02 ± 0.01
ULI wall	6.19E−02	7.24E−02	5.64E−02	6.62E−02	6.20E−02	6.38E−02 ± 0.01
Heart wall	1.10E−01	4.44E−01	9.35E−02	1.38E−01	1.23E−01	1.82E−01 ± 0.15
Kidneys^a^	2.71E+00	2.17E+00	2.55E+00	4.46E+00	1.76E+00	2.73E+00 ± 1.03
Liver	2.37E−01	1.68E−01	1.27E−01	2.17E−01	2.64E−01	2.03E−01 ± 0.05
Lungs	5.94E−02	7.10E−02	5.39E−02	6.19E−02	6.01E−02	6.13E−02 ± 0.01
Muscle	5.89E−02	6.92E−02	5.36E−02	6.20E−02	5.93E−02	6.06E−02 ± 0.01
Ovaries	6.10E−02	7.16E−02	5.54E−02	6.60E−02	6.14E−02	6.31E−02 ± 0.01
Pancreas	6.57E−02	7.60E−02	5.95E−02	7.09E−02	6.51E−02	6.74E−02 ± 0.01
Red marrow	2.17E−01	7.72E−02	1.05E−01	7.00E−02	2.25E−01	1.39E−01 ± 0.08
Osteogenic cells	1.34E+00	3.75E−01	5.73E−01	2.76E−01	1.38E+00	7.89E−01 ± 0.53
Skin	5.58E−02	6.62E−02	5.11E−02	5.85E−02	5.64E−02	5.76E−02 ± 0.01
Spleen	6.48E−02	7.48E−02	5.95E−02	7.13E−02	6.34E−02	6.68E−02 ± 0.01
Testes	5.73E−02	6.81E−02	5.24E−02	6.16E−02	5.79E−02	5.95E−02 ± 0.01
Thymus	5.84E−02	7.21E−02	5.35E−02	6.12E−02	5.92E−02	6.09E−02 ± 0.01
Thyroid	5.85E−02	6.91E−02	5.34E−02	6.04E−02	5.92E−02	6.01E−02 ± 0.01
Urinary bladder wall	2.44E−01	1.58E−01	5.78E−02	1.15E+00	1.64E−01	3.55E−01 ± 0.45
Uterus	6.13E−02	7.19E−02	5.53E−02	6.96E−02	6.14E−02	6.39E−02 ± 0.01
Total body	1.02E−01	8.76E−02	7.64E−02	8.87E−02	9.98E−02	9.09E−02 ± 0.01

^a^The range of kidney absorbed doses was 1.8−2.7 Gy/GBq; the kidney dose value of pig 4 was 4.5 Gy/GBq due to lack of amino acid infusion. The kidney absorbed dose value of pig 4 was not eliminated from the mean absorbed dose calculation

For humans after an injection of 5GBq ^177^Lu-OPS201, the highest absorbed doses are obtained for the kidneys (13.7 Gy), the osteogenic cells (3.9 Gy), the urinary bladder wall (1.8 Gy), and the liver (1.0 Gy).

In comparison to a diagnostic human study with ^68^Ga-OPS202 (^68^Ga-NODAGA-JR11) [19], spine uptake was observed and taken into account as source organ (“skeleton”) in the dose calculations. In addition, no absorbed dose to the spleen was reported in the present pig study.

Absorbed doses of ^177^Lu-OPS201 were calculated by using the mean TIACs. Pig 4 was included in the mean TIAC calculation. Although there is an overestimation of absorbed doses associated with pig 4, we did not reach any organ toxicity level. The results with and without including pig 4 were added in the relevant figure and table legends. Based on the dosimetric calculations in our pig study, the predicted absorbed doses for humans are not expected to exceed organ toxicity levels for an injection of 5–8 GBq of ^177^Lu-OPS201.

### Radioactive metabolites analyses

Urine and serial blood samples were collected for up to 12 days post-dosing and used to measure levels of radioactivity in blood and plasma, and for analysis of radioactive metabolites. No metabolites could be detected by radio HPLC both in urine excreted during anesthesia post-dosing (day 0) or in any of the blood samples.

## Discussion

In our study, we administered approximately 105 MBq (±8 MBq) ^177^Lu-OPS201 to Danish Landrace pigs. The bone and the kidneys were potential organs at risk; however, the organs with the highest absorbed doses were well below the critical organ doses [18]. Due to inter-species differences, spine uptake and lack of spleen uptake were observed in pigs in comparison to humans.

For extrapolation from animals to humans, there are empirical approaches (same biodistribution approach or body surface area (BSA)) and allometric approaches (relative mass scaling). Since our study focuses on in vivo biodistribution and dosimetry, the appropriate extrapolation method would be relative mass scaling in which the specific activity in a human organ is set equal to the specific activity in the same animal organ multiplied by the ratio of the body mass of a human and an animal. In the relative mass scaling, the calculated organ mass ratios of a human and an animal are used to scale the TIACs. In empirical approaches like the BSA method, scaling is only based on body weight [10, 20–22].

For animal studies, there are no well-accepted extrapolation methods. Since scaling most likely would decrease the values of the TIACs and, consequently, the absorbed doses, we preferred, for this study, to provide a conservative dose assessment without performing inter-species scaling.

Based on the results of our study, the amino acid infusion plays an essential role for the kidney protection. According to our data for pig 4, the lack of amino acid infusion results in a twofold increase of the absorbed doses to the kidney.

So far, in the literature, one clinical human and one preclinical mouse model study with ^177^Lu-DOTA-JR11 (OPS201) [10, 12] have been published. In the human pilot study with ^177^Lu-DOTA-JR11 (OPS201) by Wild et al. [10], before the injection of approximately 1 GBq of ^177^Lu-DOTA-JR11 (OPS201) and ^177^Lu-DOTA-octreotate, an amino acid infusion of arginine and lysine was injected to the four patients for kidney protection. A sixfold higher tumor-to-kidney ratio was observed for the antagonist ^177^Lu-DOTA-JR11 (OPS201) compared to the agonist ^177^Lu-DOTA-octreotate. The absorbed dose to the kidneys, the main organ-at-risk after treatment of neuroendocrine tumors with DOTA-labeled compounds [2], was around 50 % higher for the antagonist than for the agonist ^177^Lu-DOTA-octreotate. Although the kidney doses for ^177^Lu-DOTA-JR11 (OPS201) were reported as 1.5 times higher and the patients in this study already had grade 2 or 3 chronic renal failure, no additional decrease in tubular kidney function was reported. According to the results of their study, patients’ kidney doses should be monitored carefully after the second treatment cycle in order not to reach critical organ dose and to avoid further side effects.

Direct comparison between the results of our study and the study in humans by Wild et al. [10] may not be appropriate since the patients in this study had 2 or 3 cycles of treatment and showed already chronic renal failure. The range of kidney absorbed doses for pigs 1–3 and 5 was 1.8–2.7 Gy/GBq. Although the absorbed doses were high in our study compared to human study (overestimation related to inter-species differences), 5GBq ^177^Lu-OPS201 can be safely administered to the patients at least two times without reaching the critical organ dose for the kidney. After the second cycle of the treatment, the absorbed doses need to be analyzed to determine the injected amount of activity for further cycles. Presently, there is an ongoing clinical phase I study (EudraCT #: 2015-002867-41) which aims at finding the optimum treatment dosage in humans.

In the study by Dalm et al. [12], mice received one therapeutic injection of ^177^Lu-DOTA-JR11 (OPS201). Although the absorbed dose and TIACs values were not presented in their study, they reported comparable results to the pilot human study. The follow-up time in their mouse study was rather short especially for dosimetric and quantitative purposes. In comparison to small animals such as mice or rats, the pig model studies show more similar physical properties and biodistribution compared to human physiology. In addition, multiple blood samples can be easily drawn in pigs compared to rodents and biodistribution studies for dosimetry assessments of therapeutic agents can be carried out over a longer period compared to rodents.

## Conclusions

In general, in vivo distributions and absorbed doses of ^177^Lu-OPS201 in pigs are comparable with the literature. According to the present data, based on an animal model and extrapolation to humans, the expected radiation exposure in a human study is acceptable. However, for therapeutic applications in humans, the potential inter-species differences need to be analyzed carefully due to inconsistent spine and spleen uptake results. In the animals, ^177^Lu-OPS201 was well tolerated and produced no abnormal physiological or behavioral signs. Formation of metabolites in plasma or urine could not be observed. Consequently, the analysis of the presented experimental data provides no evidence that patients enrolled in a ^177^Lu-OPS201 study would be exposed to an unjustified overexposure of the radioactivity.

## Additional file

Additional file 1: Table S1. Radioactivity in whole blood and plasma. **Table S2.** Plasma metabolites analysis results. **Table S3.** Organ uptake values.

## Abbreviations

%A: the average percentage values corresponding to the injected radioactivity
^177^Lu: Lutetium-177
BSA: body surface area
CT: computed tomography
EOS: end of synthesis
HPLC: high-performance liquid chromatography
ITG: Isotope Technologies Garching GmbH
LLI: lower large intestine
n.c.a.: non-carrier added
NET: neuroendocrine tumor
PEEP: positive end expiratory pressure
PRRT: peptide receptor radionuclide therapies
SD: standard deviation
SST2: somatostatin receptor
TACs: time-activity curves
TIACs: organ-specific time-integrated activity coefficients
ULI: upper large intestine
VOI: volume of interest
WB: whole-body

## Footnotes

1 In the literature, often also called “DOTA-JR11”.

2 In the literature, often also called “NODAGA-JR11”.
