# Supplementary material for: 177Lu-OPS201 targeting somatostatin receptors: in vivo biodistribution and dosimetry in a pig model
Source: EJNMMI Res. 2016 Jun 13;6:50. doi: 10.1186/s13550-016-0204-9 (PMC4906090; doi:10.1186/s13550-016-0204-9)
Supplement: Additional file 1: Table S1. — Radioactivity in whole blood and plasma. Table S2. Plasma metabolites analysis results. Table S3. Organ uptake values. [file 13550_2016_204_MOESM1_ESM.pdf]

^{177}Lu-OPS201 Targeting Somatostatin Receptors: In Vivo Biodistribution and Dosimetry in a Pig Model

Supplementary file

Beykan Seval[1], Dam Jan S.[2], Eberlein Uta[1], Kaufmann Jens[3], Kjærgaard Benedict[4], Jødal Lars[2], Bouterfa Hakim[3], Bejot Romain[3], Lassmann Michael[1], Jensen Svend Borup[2, 5]

1: Department of Nuclear Medicine, University of Würzburg, Würzburg, Germany

2: Department of Nuclear Medicine, Aalborg University Hospital, Denmark

3: Octreopharm Science GmbH, Berlin, Germany

4: Biomedical Research Laboratory, Department of Clinical Medicine, Aalborg University Hospital Denmark

5: Department of Chemistry and Biochemistry, Aalborg University, DK-9000 Aalborg, Denmark

Supplementary Table 1. Radioactivity in whole blood and plasma

Animal IA: OG-20140920-P1

Time post dosing (min)	Whole blood		Plasma	
	Bq/mL*	%IA/mL**	Bq/mL*	%IA/mL**
1.40	16156	0.01517	22058	0.02072
2.15	13592	0.01277	17445	0.01638
3.00	10943	0.01028	14867	0.01396
5.00	7720	0.00725	10170	0.00955
10.02	4628	0.00435	6110	0.00574
20.03	2684	0.00252	3712	0.00349
30.17	1950	0.00184	2787	0.00262
50.03	1400	0.00132	1937	0.00183
75.16	1054	0.00100	1389	0.00131
99.5	884	0.00084	1173	0.00111
200.1			744	0.00071
300.0	396	0.00038	528	0.00051
3180	84	0.00010	134	0.00016
6060	58	0.00009	94	0.00014
8892	29	0.00005	54	0.00010
17610	4	0.00001	9	0.00003

Animal IA: OG-20140920-P2

Time post dosing (min)	Whole blood		Plasma	
	Bq/mL*	%IA/mL**	Bq/mL*	%IA/mL**
1.18	24986	0.02443	39017	0.03815
1.22	20887	0.02078	32837	0.03266
3.20	12499	0.01243	18324	0.01823
5.05	9373	0.00933	13686	0.01362
10.05	5494	0.00547	8304	0.00827
20.00	3153	0.00314	4860	0.00484
30.10	2278	0.00227	3484	0.00347
50.00	1686	0.00168	2340	0.00234
75.02	1193	0.00119	1834	0.00183
100.05	939	0.00094	1426	0.00143
200.05	659	0.00067	1027	0.00104
300.0	498	0.00051	672	0.00068
3030	101	0.00012	162	0.00020
5910	64	0.00010	99	0.00015
8730	37	0.00007	62	0.00012
17400	6	0.00002	13	0.00005

* decay-corrected to the time of sampling

** %IA/mL denotes percentage injected activity per volume of blood

Animal IA: OG-20140920-P3

Time post dosing (min)	Whole blood		Plasma	
	Bq/mL*	%IA/mL**	Bq/mL*	%IA/mL**
0.35	42509	0.04030	55934	0.05302
1.22	35041	0.03322	48037	0.04554
2.13	26473	0.02510	36024	0.03415
5.00	16265	0.01542	21724	0.02060
10.34	10020	0.00951	13555	0.01286
19.56	6587	0.00625	9026	0.00857
29.44	4965	0.00472	6775	0.00644
49.00	3310	0.00315	4687	0.00446
82.20	2087	0.00199	3379	0.00322
100.14	1838	0.00176	3643	0.00348
199.18	1452	0.00140	2013	0.00194
300.0	1071	0.00104	1777	0.00172
3360	138	0.00017	159	0.00019
6180	43	0.00006	69	0.00010
14880	10	0.00003	12	0.00003

Animal IA: OG-20140920-P4

Time post dosing (min)	Whole blood		Plasma	
	Bq/mL*	%IA/mL	Bq/mL*	%IA/mL**
1.00	29160	0.02581	38536	0.03410
1.15	27344	0.02420	34930	0.03092
2.05	21540	0.01907	28053	0.02483
5.05	13905	0.01231	18353	0.01625
10.02	8458	0.00749	11094	0.00983
19.58	5007	0.00444	6540	0.00580
29.48	3651	0.00324	4878	0.00433
49.55	2713	0.00241	3450	0.00306
74.56	2058	0.00183	2641	0.00235
99.45	1673	0.00149	2136	0.00190
225.0	1085	0.00098	1447	0.00130
300.0	829	0.00075	1102	0.00100
3360	168	0.00019	202	0.00023
6180	93	0.00013	122	0.00017
14880	11	0.00003	12	0.00003

* decay-corrected to the time of sampling

** %IA/mL denotes percentage injected activity per volume of blood

Animal IA: OG-20140920-P5

Time post dosing (min)	Whole blood		Plasma	
	Bq/mL*	%IA/mL**	Bq/mL *	%IA/mL**
0.40	43410	0.04476	68287	0.07040
0.57	31774	0.03276	48412	0.04991
1.56	14945	0.01541	29290	0.03020
4.58	8304	0.00856	13910	0.01435
10.49	6029	0.00622	8459	0.00873
20.06	3825	0.00395	5395	0.00557
29.22	2821	0.00291	4134	0.00427
49.44	1827	0.00189	2756	0.00285
76.00	1499	0.00155	2036	0.00211
100.0	1138	0.00118	1726	0.00179
206.1	758	0.00079	1161	0.00121
293.0	574	0.00060	835	0.00088
3360	127	0.00016	171	0.00022
6000	72	0.00012	105	0.00017
14700	12	0.00004	14	0.00004

* decay-corrected to the time of sampling

** %IA/mL denotes percentage injected activity per volume of blood

Supplementary Table 2. Plasma metabolites analysis results

Animal IA	Nominal sampling time	Radiochemical purity (HPLC)
Pig 1	5 min	97.4%
	30 min	-
	50 min	96.6%
	100 min	97.8%
	200 min	-
	300 min	96.7%
	2 h	96.5%
	4 h	95.6%
	6 h	99.0%
	12 h	98.8%
Pig 2	5 min	98.9%
	30 min	97.5%
	50 min	-
	100 min	94.8%
	200 min	96.5%
	300 min	96.7%
	2 h	98.0%
	4 h	99.7%
	6 h	98.5%
	12 h	99.1%
Pig 3	5 min	100%
	30 min	100%
	50 min	100%
	100 min	100%
	200 min	100%
	300 min	100%
	2 h	99.9%
	4 h	100%
	10 h	100%

Pig 4	5 min	100%
	30 min	100%
	50 min	100%
	100 min	100%
	200 min	100%
	300 min	100%
	2 h	100%
	4 h	100%
	10 h	98.5%
Pig 5	5 min	100%
	30 min	100%
	50 min	100%
	100 min	100%
	200 min	99.8%
	300 min	100%
	2 h	99.9%
	4 h	99.9%
	10 h	99.9%

The results of denatured plasma samples were analyzed by HPLC for the detection of radioactive metabolites.

Supplementary Table 3. Organ uptake values

Time<br>h	WB<br>%IA	Liver<br>%IA	R. Kidney<br>%IA	L. Kidney<br>%IA	Bladder<br>%IA	Heart<br>%IA	Spine<br>%IA
Pig 1							
0.7	100.000	6.981	1.999	2.582	0.223	0.883	3.047
1.9	96.343	6.767	2.515	2.922	0.368	1.151	3.431
3.0	95.535	6.725	2.700	3.150	0.690	0.804	3.439
54.0	50.974	2.646	2.780	2.796	1.046	0.287	2.157
101.4	29.934	1.707	1.775	1.772	0.000	0.207	1.448
148.9	18.522	1.114	1.142	1.108	0.210	0.099	1.021
293.7	2.414	0.021	0.434	0.352	0.000	0.000	0.002
Pig2							
0.5	100.000	3.211	1.914	2.245	2.793	0.680	3.544
1.6	86.080	2.963	2.230	2.602	1.361	0.477	3.779
4.5	86.334	2.741	2.356	2.653	1.265	0.504	4.208
51.6	45.575	2.260	1.988	2.210	0.005	0.304	2.543
99.1	27.356	1.298	1.277	1.519	0.010	0.190	1.414
146.3	17.888	0.817	0.851	0.977	0.083	0.117	1.163
242.5	2.315	0.034	0.261	0.309	0.000	0.007	0.069
Pig3							
0.9	100.000	4.377	1.104	2.118	0.063	1.232	2.280
2.0	79.024	3.774	1.353	2.307	0.065	1.035	2.413
3.8	77.890	3.021	1.929	3.653	0.015	0.804	3.198
56.6	34.342	1.297	2.052	2.477	0.029	0.187	1.375
102.9	19.963	0.976	1.226	1.569	0.001	0.086	0.909
248.1	2.778	0.112	0.332	0.364	0.000	0.000	0.065
Pig4							
0.5	96.283	7.869	2.944	2.698	8.604	0.685	2.181
1.7	98.664	7.652	3.223	3.067	14.207	0.471	2.396
2.8	100.000	7.346	3.454	3.255	16.182	0.391	2.513
54.6	42.530	2.056	2.445	3.838	0.610	0.191	1.925
103.0	25.839	1.356	1.645	1.582	0.461	0.099	1.007
248.2	4.029	0.171	0.490	0.487	0.002	0.002	0.164
Pig5							
0.6	100.000	7.507	1.720	1.589	0.104	0.716	2.739
1.8	91.449	7.596	1.853	1.754	0.055	0.648	3.467
3.0	89.169	7.531	2.064	1.850	0.042	0.541	3.389
50.7	46.296	1.932	1.615	2.234	0.627	0.244	2.478
101.0	28.021	1.618	0.977	1.007	0.200	0.189	1.476
245.5	5.751	0.442	0.355	0.292	0.000	0.028	0.434
